# Functional analysis of enhancer elements regulating the expression of the *Drosophila* homeodomain transcription factor DRx by gene targeting

**DOI:** 10.1186/s41065-021-00210-z

**Published:** 2021-11-05

**Authors:** Christine Klöppel, Kirsten Hildebrandt, Dieter Kolb, Nora Fürst, Isabelle Bley, Ruth-Jessica Karlowatz, Uwe Walldorf

**Affiliations:** 1 Developmental Biology, Saarland University, Building 61, 66421 Homburg/Saar, Germany; 2 Present address: Genetics/Epigenetics, Saarland University, Building A2.4, 66123 Saarbrücken, Germany; 3 Present address: Research Institute Children’s Cancer Center Hamburg, Building N63, Martinistr. 52, 20251 Hamburg, Germany

**Keywords:** Drosophila retinal homeobox (DRx), Transcription factor, Enhancer, Gene targeting

## Abstract

**Background:**

The *Drosophila* brain is an ideal model system to study stem cells, here called neuroblasts, and the generation of neural lineages. Many transcriptional activators are involved in formation of the brain during the development of *Drosophila melanogaster*. The transcription factor *Drosophila* Retinal homeobox (DRx), a member of the 57B homeobox gene cluster, is also one of these factors for brain development.

**Results:**

In this study a detailed expression analysis of DRx in different developmental stages was conducted. We show that DRx is expressed in the embryonic brain in the protocerebrum, in the larval brain in the DM and DL lineages, the medulla and the lobula complex and in the central complex of the adult brain. We generated a DRx enhancer trap strain by gene targeting and reintegration of Gal4, which mimics the endogenous expression of DRx. With the help of eight existing enhancer-Gal4 strains and one made by our group, we mapped various enhancers necessary for the expression of DRx during all stages of brain development from the embryo to the adult. We made an analysis of some larger enhancer regions by gene targeting. Deletion of three of these enhancers showing the most prominent expression patterns in the brain resulted in specific temporal and spatial loss of DRx expression in defined brain structures.

**Conclusion:**

Our data show that DRx is expressed in specific neuroblasts and defined neural lineages and suggest that DRx is another important factor for *Drosophila* brain development.

**Supplementary Information:**

The online version contains supplementary material available at 10.1186/s41065-021-00210-z.

## Background

Rx genes belong to a highly conserved gene family coding for transcription factors with a paired-like homeodomain [1]. They were first identified in *Xenopus* [2] and mice [3, 4] as essential regulators of vertebrate eye development. Rx genes were also identified in chickens, medaka, zebrafish and humans and are expressed in the eye and forebrain (reviewed in [5]). Shortly after the identification of the first Rx genes in vertebrates, an Rx gene was also identified in *Drosophila* [6]. Unexpectedly the *Drosophila* Rx gene, called DRx, was found to have no function in eye development in *Drosophila* but was expressed in the brain from the embryonic to the adult stages [6, 7].

The *Drosophila* brain is formed by 108 bilaterally arranged lineages [8–11]. Each lineage derives from neuroblasts, which are stem cells that could divide symmetrically as shown for neuroblasts in the inner proliferation centre of the optic lobe [12], but mostly divide asymmetrically and thereby through self-renewal generate a further neuroblast and a neuronal precursor cell, the ganglion mother cell (GMC). The GMC then divides symmetrically and produces two neurons. Through this mode of division, the neuroblast produces embryonic lineages of primary neurons (reviewed in [13]). This type of division is typical for type I neuroblasts, that build most of the cell lineages in the embryonic brain. In contrast to type I neuroblasts, eight type II neuroblasts generate intermediate neural progenitor cells (INPs) that divide several times to generate GMCs, which in turn divide into two neurons [14–16], thereby generating larger lineages. Moreover, it was shown, that these type II neuroblasts and the corresponding lineages are already present in later stages of embryonic brain development [17, 18]. At the end of embryogenesis, most neuroblasts undergo a period of quiescence, resume their division during the early larval stage and continue dividing up to the late pupal stages (reviewed in [19]). In contrast, four mushroom body neuroblasts (MBNBs) generate 30–40 cells in the embryo and continuously divide up to the late pupal stage [20]. In the postembryonic phase secondary neurons develop that make up 90% of the adult neurons. In the larval brain, all neuroblasts generate larger lineages compared with the embryonic brain, and type I lineages produce a progeny of 100 neurons, the eight type II lineages even up to 400 neurons (reviewed in [21]). Six of the eight type II lineages are located in the dorsomedial region (DM1–6), and the other two lineages are located in the dorsolateral region (DL1, 2) of the larval brain [22, 23].

In the embryonic brain, DRx expression is detected in some neuroblasts, including the four mushroom body neuroblasts [20, 24]. Here, DRx promotes cell growth, proliferation and survival of mushroom body neuroblasts [24]. In the larval brain, DRx is expressed in the optic lobes [25], processing centres for the visual information [26], and later in the ellipsoid body, a part of the central complex of the adult brain [7]. The ellipsoid body that controls specific locomotor skills, such as walking and flight activities [27–29], but also visual guidance, orientation and turning behaviours [30–35], is missing in DRx mutant flies [7]. Some data concerning the function of DRx were identified through genome-wide analyses. An RNAi screen in larval neuroblasts and INPs showed that the downregulation of DRx causes an underproliferation phenotype [36]. Transcriptional profiling identified DRx as a factor expressed in larval type I but not type II neuroblasts [37]. Through the analysis of the transcriptomes of lineage-specific neuroblasts, it was further shown that DRx is expressed in all larval mushroom body neuroblasts and in one of the four antennal lobe neuroblasts [38].

More recently, it was shown that DRx is also one of several transcription factors that are expressed in type II neuroblasts in the embryonic brain and important for progenitor cell proliferation leading to an expansion of the brain region compared with the ventral nerve cord [39]. Homeodomain transcription factors such as Orthopedia (Otp) [40, 41] and Homeobrain (Hbn) [42, 43] belong to these factors and are together with DRx encoded next to each other in the 57B region on the second chromosome [42]. Mutants of all these factors alone or in combination show a reduction in neuroblasts and the proliferation of their daughter cells. Upon misexpression, all of them can drive forward proliferation in the ventral nerve cord and can even reprogramme wing disc cells into brain neural progenitors [39]. Due to the complex expression pattern of DRx during all stages of brain development, DRx seems to play an important role not only in mushroom body and type I and II neuroblast development but also during the differentiation and generation of the respective structures like building of the adult ellipsoid body.

One major question concerns how the complex expression patterns of DRx are established and maintained over time. It is well accepted that the expression of genes in specific expression domains in cells or tissues is regulated by sets of regulatory elements, which include enhancers that can act over large distances. To analyse such elements in animals, transgenic reporter gene assays are usually performed using lacZ or GFP as reporter genes (reviewed in [44]). In *Drosophila,* the *fushi tarazu* (*ftz*) enhancer was the first enhancer identified by this method and serves as a classical example [45]. Later, many additional enhancers were identified in a similar manner. During the course of the *Drosophila* genome project, further systematic attempts were conducted to identify enhancers of genes with a known expression or function in the adult brain [46]. To achieve this goal, overlapping DNA pieces of 3 kb located upstream, downstream or in introns of 925 genes with known expression in the brain were cloned in front of a Gal4 gene. Following the generation of more than 5000 transgenic fly strains, the expression patterns of putative enhancers were analysed using reporter genes in different developmental stages and tissues [47–49]. Integration of the constructs into the same chromosomal position allowed a direct comparison of the enhancer activities, avoiding position effects. Among these strains were also some from the genomic region of DRx that were available from the Bloomington Drosophila Stock Center. In a complementary effort, additional strains were generated [50] and are available from the Vienna Drosophila Research Center. In a recent publication, a DRx enhancer driving the expression in the outer proliferation centre of the developing optic lobe was identified [51]. Analysis of all available strains to define enhancer regions of the DRx gene would provide a definitive step toward understanding the complex regulation of the gene, but a functional definition of such regulatory elements through transcription factor DNA interactions and mutant enhancer variants might be one major goal for the future (reviewed in [52]).

In this paper we focused on the expression of DRx in embryonic and postembryonic stages and analysed enhancer elements regulating the expression of DRx during brain development. This analysis shows that DRx is expressed in all type II lineages of the larval brain, some type I lineages and in the medulla and lobula plate. In type II lineages expression is seen in some INPs, in GMCs and neurons, but not in glial cells. Putative regulatory elements of DRx were analysed with the help of several Gal4 strains harbouring various enhancer fragments from the upstream regions as well as the two largest intron regions of DRx. We identified several regulatory modules responsible for the complex expression of DRx in the embryonic, larval and adult brains. By gene targeting we generated a new DRx strain carrying a deletion of the coding part of the first exon, including the ATG, and by reintegration of Gal4 at this position, a DRx enhancer trap strain was generated and analysed. In the final analysis, three enhancer regions driving prominent expression in the type II lineages and the optic lobe were individually deleted by gene targeting and their effects on the expression of DRx were analysed. Our findings imply an important function of DRx in various processes of *Drosophila* brain development.

## Results

### Expression of DRx during brain development

The expression pattern of DRx has been analysed in the embryo by in situ hybridization [6] after which primarily an anti-DRx antibody generated by us was used to analyse the DRx phenotype in the adult brain [7]. More detailed analyses of earlier processes focused on the function of DRx during mushroom body development [20, 24] and optic lobe development [25]. Here, we wanted to analyse the expression of DRx during development in more detail, focusing on unexplored expression domains, especially in the larval brain, and to determine the enhancers responsible for the temporal and spatial expression patterns of DRx.

**Fig. 1:**
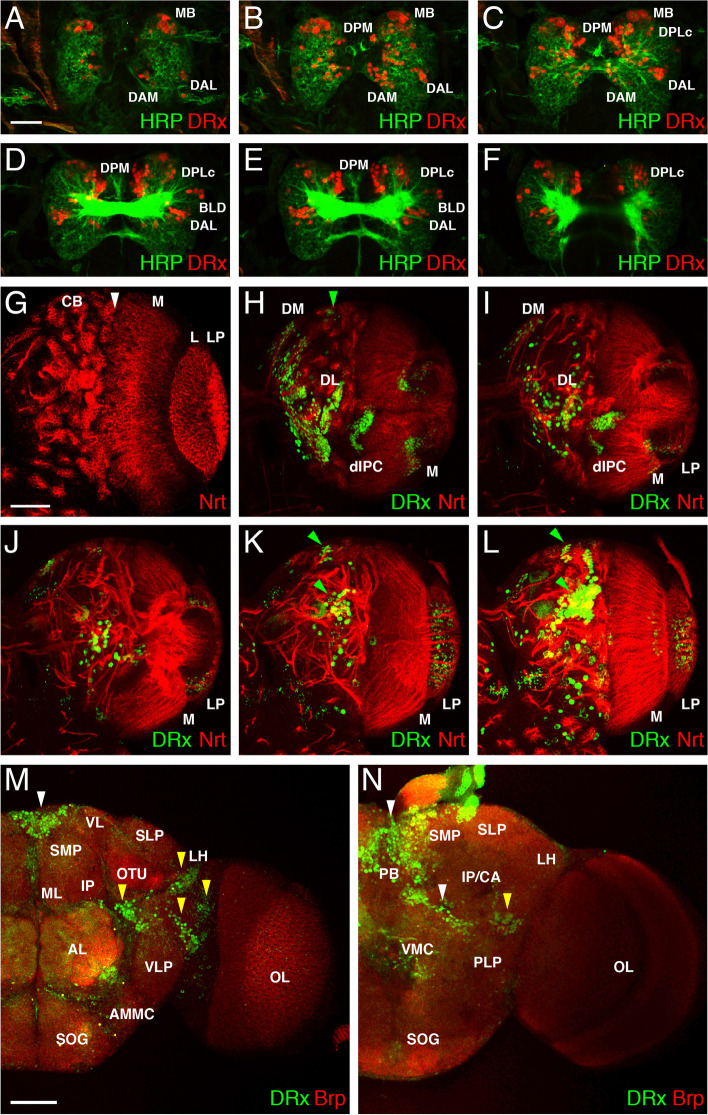
DRx expression during *Drosophila* development. Laser confocal images of *Drosophila* embryonic, larval and adult brains. **A-F** Sections of an embryonic brain at stage 16 from the dorsal to the ventral side using HRP (green) and DRx (red). Major expression domains are indicated from the dorsal to ventral region. Abbreviations: BLD, basal lateral dorsal; DAL, dorsal anterior lateral; DAM, dorsal anterior medial; DPLc, dorsal central lateral; DPM, dorsal posterior medial; MB, mushroom body. **G** Ventral view of a right larval brain hemisphere (L3) to highlight the main structures using anti-Nrt staining. The white arrowhead marks the border between the central brain and the optic lobe. CB, central brain with type I and II lineages; M, medulla; L, lamina; LP, lobula plate. **H-L** Dorsal to ventral sections of a right larval brain hemisphere stained with anti-DRx (green) and anti-Nrt (red). The largest expression domains are indicated: the dorsomedial domain (DM), dorsolateral domain (DL), dorsal inner proliferation centre (dIPC), medulla (M) and lobula plate (LP). Additional smaller domains are indicated by green arrowheads. **M, N** Two different focal planes of the right side of an adult brain showing the expression of DRx in green and Brp in red. **M** In the more anterior focal plane, DRx expression can be observed in a discrete central domain (white arrowhead) and lateral domains (yellow arrowheads). **N** Expression in a more posterior focal plane is evident in a central domain close to the protocerebral bridge (PB) (white arrowheads) and dorsal to the posterior-lateral protocerebrum (PLP) (yellow arrowhead). Abbreviations: AL, antennal lobe; AMMC, antenna-mechanosensory and motor centre; CA, calyx (mushroom bodies); IP, inferior protocerebrum; LH, lateral horn; ML, medial lobe (mushroom bodies); OL, optic lobe; OTU optic tubercle; PB, protocerebral bridge; PLP, posterior-lateral protocerebrum; SLP, superior-lateral protocerebrum; SMP, superior-medial protocerebrum; SOG, subesophageal ganglion; VL, vertical lobe (mushroom bodies); VMC, ventro-medial cerebrum; VLP, ventro-lateral protocerebrum. (Scale bars: 50 μm; A-F like A, G-L like G, N like M)

To analyse the embryonic brain expression of DRx we used in addition HRP, a general neuronal marker [53], to highlight the brain structure. In specific domains of different brain sections labelled according to the order of emergence from dorsal to ventral, DRx expression was visible in the brain (Fig. 1A-F) These domains were assigned to lineage groups according to [54, 55]. Large mushroom body neuroblasts and progenitor cells were in domain MB (Fig. 1A-C). The DAM (dorso anterior medial) domain was located medially in close proximity to the brain commissure, and the DAL (dorso anterior lateral) domain was located in a lateral region (Fig. 1A-E). Strong expression was observed in the DPM (dorso posterior medial) domain (Fig. 1B-E). Other domains were DPLc (dorsal central lateral) (Fig. 1C-F) and BLD (basal lateral dorsal) (Fig. 1D, E). In particular, the expression in domain DPM was very pronounced; here, DRx was more broadly expressed than Hbn, another factor expressed in that area [39, 43].

The expression of DRx in the larval brain was again analysed in combination with a neuronal marker, here Neurotactin (Nrt) [56] which is expressed in many postembryonic neurons and their axons. The larval brain is subdivided into the central brain regions (CB) and the optic lobes, and the visual processing centres consist of the medulla (ME), lamina (L) and lobula plate (LP) [57, 58]. Nrt staining of the right hemisphere of the larval brain (L3) showed these main structures in detail (Fig. 1G). Cells of the optic lobe derive from an outer proliferation centre (OPC) generating the medulla and lamina and an inner proliferation centre (IPC) generating mainly cells of the lobula complex [58]. In the central brain region (CB) with the type I and type II lineages, a prominent expression of DRx was visible in the dorsomedial region (DM) and in the dorsolateral region (DL) where the type II lineages are located (Fig. 1H, I). Additional domains were found in type I Iineages of the central brain (Fig. 1H, green arrowhead) [59, 24]. In the optic lobe region, expression was visible in the dorsal inner proliferation centre (dIPC) (Fig. 1H, I) and in the medulla (M) (Fig. 1H) (see also [25]). In more medial and ventral sections, DRx was also expressed in lobula plate (LP) neurons (Fig. 1I-L). In ventral sections, additional regions of the central brain showed DRx expression (Fig. 1K, L, green arrowheads).

The expression of DRx in the adult brain was analysed in combination with an antibody against Bruchpilot (Brp) which labels synapses and can be used to mark the neuropile [60]. In a more anteriorly located focal plane of the adult brain, DRx expression was detected in a central area of the brain (Fig. 1M, white arrowhead). Additionally, DRx expression was detected in more laterally located regions ventral of the optic tubercle (OTU) and lateral horn (LH), dorsal of the ventro-lateral protocerebrum (VLP) and close to the optic lobe (Fig. 1M, yellow arrowheads). In a more posterior focal plane, prominent expression was seen in a central region near the protocerebral bridge (PB) (Fig. 1N, white arrowheads) and in a lateral region dorsal to the posterior-lateral protocerebrum (PLP) (Fig. 1N, yellow arrowhead).

**Fig. 2:**
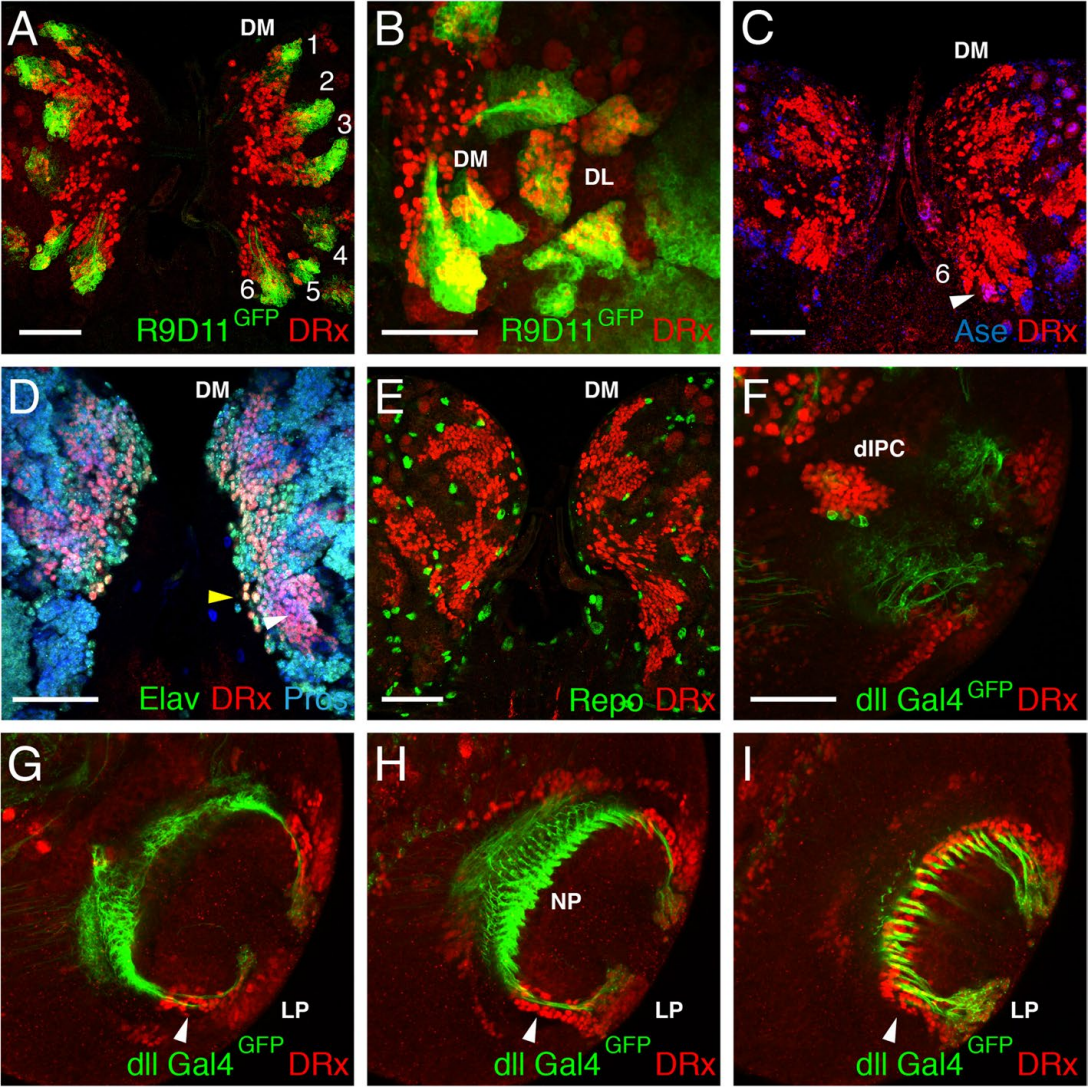
Cell type identification of DRx-expressing cells in the larval brain. **A-E** Larval brains with the central brain regions. **A** The earmuff R9D11 reporter (R9D11-mCD8-GFP, green) highlights the proximal parts of the six DM lineages (1–6) starting from the INPs. DRx expression (red) is visible in the more central and distal parts of these lineages. **B** Expression of the earmuff R9D11 reporter (R9D11-mCD8-GFP, green) also highlights the two DL lineages adjacent to the DM lineages. Here, DRx (red) is expressed in more cells of the lineages compared with the neighbouring DM lineages. **C** Ase expression (blue) marks INPs of type II lineages. Coexpression with DRx (red) was detected in only a few cells, mostly evident in DM6 (white arrowhead). **D** Expression of DRx (red) in combination with Pros (blue) and Elav (green) indicates DRx expression in GMCs (Pros^+^, Elav^−^, white arrowhead) and neurons (Pros^+^, Elav^+^, yellow arrowhead). **E** DRx expression (red) in combination with the glial cell marker Repo (green) shows no coexpression. **F-I** Sections of a larval brain hemisphere focusing on the lobula complex. DRx expression (red) is shown with dll Gal4-mCD8::GFP (green). Abbreviations: DL, dorsolateral lineages; DM, dorsomedial lineages; dIPC, dorsal inner proliferation centre; LP, Lobula plate; NP, neuropile. (Scale bars: 50 μm, 2B 20 μm)

For a more precise characterization of the DRx expression in the larval brain with respect to brain structures, lineages and cell types, we used specific markers. Here, type II lineages were of great interest, since structures of the central complex in the adult brain are derived from these lineages and DRx expression is necessary for some structures of the central complex [7]. To assign DRx expression in the dorsomedial region to DM lineages, we used the Gal4 line Erm-Gal4-R9D11 [46], which shows specific expression in the proximal parts of the DM lineages in INPs and GMCs [22, 61, 62]. The Gal4 line was crossed with UAS-CD8::GFP (membrane labelling) to visualize the expression. DRx was expressed in all DM lineages adjacent to Erm-Gal4 in the more distal parts of each lineage (Fig. 2A). DRx expression in the DL lineages was analysed in a similar way; here, DRx was visible in DL1 und DL2, but compared with the neighbouring DM lineages, DRx was expressed in more cells of these lineages (Fig. 2B). To address the question in which cell types DRx is expressed within the DM lineages we used specific cell type markers. Dpn, a marker for neuroblasts, showed no colocalization with DRx (data not shown), as expected from the previous results. When we used Asense (Ase) expression as a marker for INPs, here some cells in each lineage showed colocalization with DRx, but this number was clearly increased in the DM6 lineages (Fig. 2C, white arrowhead). To discriminate between GMCs and neurons we used Prospero (Pros) and Elav as markers. Pros expression occurs in the cytoplasm in neuroblasts and in the nucleus in GMCs as well as in postmitotic neurons [63, 64], whereas Elav is only expressed in postmitotic neurons [65, 66]. Therefore, GMCs are Pros positive and Elav negative, and neurons are Pros and Elav positive. This staining in combination with DRx shows that DRx is expressed in GMCs (Fig. 2D, white arrowhead) as well as in neurons (Fig. 2D, yellow arrowhead). In contrast to Pros and Elav, the glial cell marker Reversed polarity (Repo) [67, 68] did not show colocalization with DRx (Fig. 2E). In summary this analysis showed that DRx expression in DM lineages did not occur in neuroblasts and glia cells but in some INPs and mainly in GMCs and neurons.

The expression of DRx in the medulla was not analysed further since DRx was previously shown to be expressed in the posterior arms of the outer proliferation centre [25, 51]. However, in addition to its expression in the medulla, DRx was also expressed in the lobula complex. To analyse lobula plate expression more specifically, we used the marker dll-Gal4 to visualize the neurons of the lobula plate and their axonal projections. Sections of a brain hemisphere showed DRx expression in the dorsal inner proliferation centre (dIPC) (Fig. 2F) and the lobula plate (LP) (Fig. 2G-I, white arrowheads). DRx is prominently expressed in cells surrounding the axonal projections of the lobula plate to the neuropil.

### Generation of a DRx strain with reintegration of Gal4 in the DRx locus

**Fig. 3:**
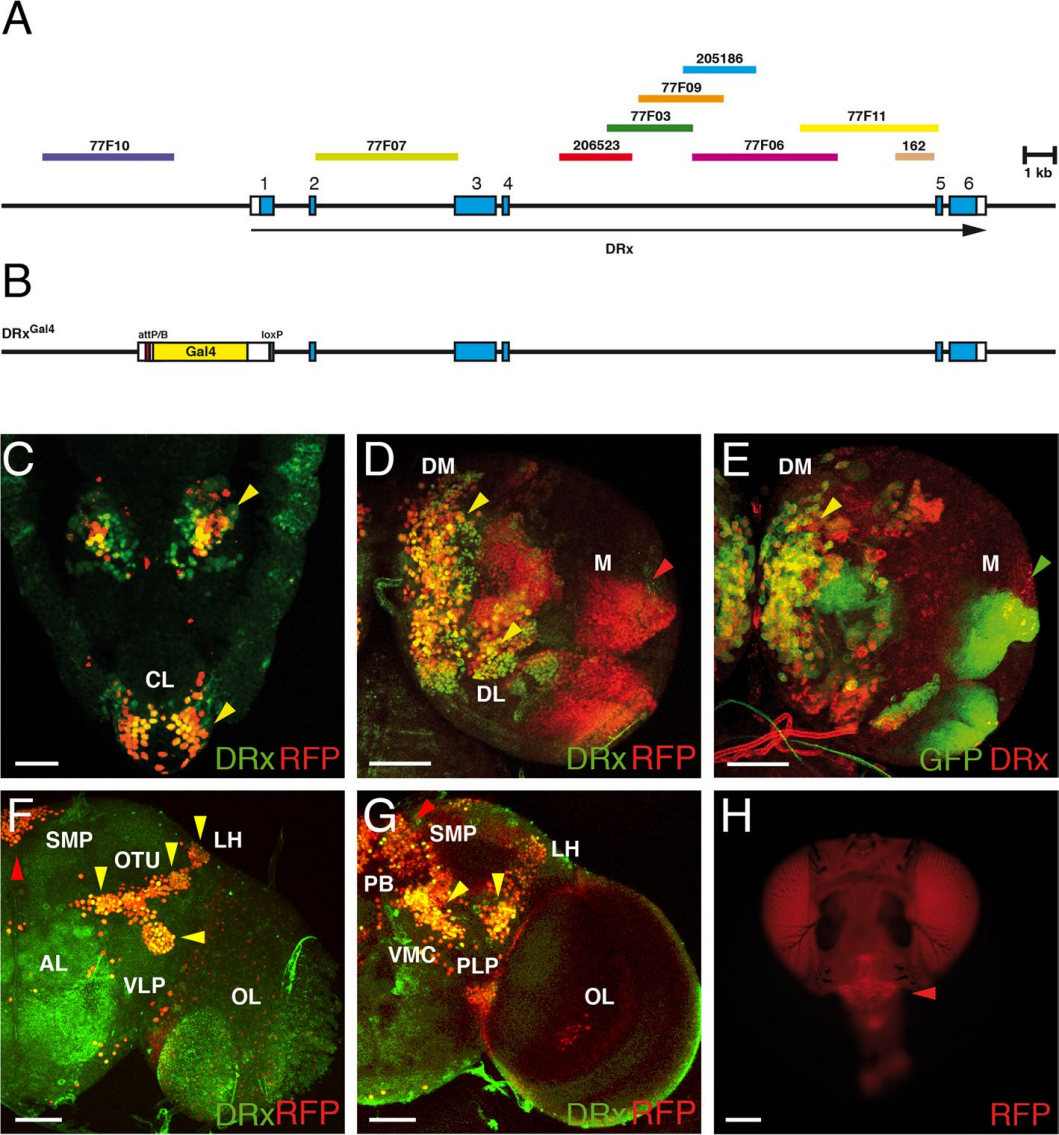
Generation and expression of the DRx^Gal4^ strain. **A** The genomic organization of the DRx locus is shown with the positions of the six exons specific for the transcript variant DRx1 [6]. Noncoding regions are indicated by white boxes, and coding regions are indicated by blue boxes. The location of fragments used for the later analysis of enhancer activities is also indicated. **B** The genomic organization of the DRx^Gal4^ strain is shown. Here, the region upstream of the ATG in exon 1 up to sequences shortly downstream of the exon 1 donor splice site has been deleted and replaced by Gal4 (yellow) flanked by an attP/B site (red) and a loxP site (green). **C-G** Laser confocal images showing the expression of the DRx^Gal4^ strain in different developmental stages visualized using a UAS-H2B-mRFP1 strain and a UAS-mCD8::GFP strain. **C** In a stage 16 embryo (the anterior end of the embryo is pointing downward), DRx expression is shown in green and DRx^Gal4^ dependent marker RFP expression is shown in red. Strong coexpression of the nuclear markers RFP and DRx can be observed in the embryonic brain and clypeolabrum (CL) (yellow arrowheads). **D** In the right hemisphere of a third instar larval brain, DRx and DRx^Gal4^ marker coexpression in the type II lineages (DM, DL) is shown (yellow arrowheads). In the medulla (M), only the RFP marker is expressed (red arrowhead). **E** Coexpression in the DM lineages is also visible using GFP as a marker (yellow arrowhead); in the medulla, again only GFP is expressed. **F, G** Additionally, in the adult brain, coexpression of DRx and RFP is detectable in an anterior focal plane (**F**) and a posterior focal plane (**G**) in most regions (yellow arrowheads) except for the protocerebral bridge, here more RFP marker expression is visible (red arrowheads) Abbreviations: AL, antennal lobe; LH, lateral horn; OL, optic lobe; OTU optic tubercle; PB, protocerebral bridge; PLP, posterior-lateral protocerebrum; SMP, superior-medial protocerebrum; VMC, ventro-medial cerebrum; VLP, ventro-lateral protocerebrum. **G** The RFP marker expression of the DRx^Gal4^ is also apparent in living flies in the clypeus (red arrowhead), a structure that is missing in DRx mutants [7]. (Scale bars: C, 25 μm; D-F, 50 μm; G, 100 μm)

For future experiments it would be beneficial to develop an DRx enhancer trap strain that recapitulates almost the complete DRx expression pattern during development. Under ideal circumstances all DRx enhancers should activate Gal4 integrated in the locus to use such a strain for overexpression, downregulation or rescue experiments. To follow up on this idea, we used the gene targeting vector pTV^cherry^ [69], which is suitable for this experimental design. With this vector, it is possible to generate a small deletion in DRx to inactivate the gene by gene targeting and at the same time integrate an attP site into the locus. With the use of a reintegration vector it is then possible to integrate Gal4 into the locus using the attP site. The DRx gene has six exons spanning a region of approximately 20 kb (Fig. 3A). We decided to delete a region of 394 bp starting 10 bp upstream of the ATG of the first intron including the donor splice site. This deletion removed the first 123 amino acids of DRx. We amplified and cloned two 2.7 kb homologous regions flanking the area to be deleted into the pTV^cherry^ vector, created transgenic fly lines and mapped their chromosomal position. For the targeting event by homologous recombination, we used a strain carrying an integration of the construct on the third chromosome. Among 15,851 flies representing the offspring of our gene targeting crosses, we identified 25 red eyed flies, resulting in a gene targeting frequency of 1/634. Some of these flies were balanced and analysed by PCR to verify that the homologous recombination event was correct. In one of the resulting DRx targeting strains, we initially called DRx^KO^ sequences encoding the ATG and the first 123 amino acids were replaced by a cassette including a white marker, loxP sites and an attP sequence [69]. Unexpectedly, we obtained homozygous viable flies after balancing, whereas pre-existing DRx alleles were lethal at the pupal stage. Using the loxP sites, we removed the white gene, leaving back an attP sequence in the DRx locus. Additionally, this strain without the white gene was homozygous viable. We therefore analysed whether DRx was expressed in this strain and discovered its normal expression in homozygous embryos and larvae (data not shown). This phenomenon may have been due to the use of an alternatively used downstream ATG, resulting in an N-terminal truncated DRx protein that was detected by the antibody and seemed to be functional. Even if our strain was obviously not a DRx mutant strain, we followed up our initial plan to reintegrate Gal4 in the DRx locus at the attP position with the help of the reintegration vector RIV^Gal4^ [69]. After selection of the correct transgenic flies, the white marker was again removed using the flanking loxP sites so that in the final fly strain, which we called DRx^Gal4^, Gal4 and some adjacent sequences had replaced the deleted exon sequences of DRx (Fig. 3B).

To analyse the expression of DRx^Gal4^ we visualized the Gal4 expression with the help of fluorescent markers (H2B-mRFP1 and mCD8::GFP) [70] in wild-type embryos, larvae and adults (Fig. 3C-G). In the embryo, coexpression was detected in the clypeolabrum (CL) and some areas of the brain (Fig. 3C, yellow arrowheads). In the brain, some cells only expressed DRx or the RFP marker, which might be due a temporal delay in the expression of the marker RFP compared with the DRx protein, conversely, DRx expression might vanish and RFP marker expression persist for some time, an effect which is usually observed using reporter gene expression. The expression of DRx and the markers RFP and GFP showed strong coexpression in the DM and DL lineages of the larval brain (Fig. 3D, E, yellow arrowheads), whereas much more marker expression compared with DRx expression was seen in the medulla (M) (Fig. 3D, red arrowhead; Fig. 3E, green arrowhead). In the adult brain, coexpression was also observed in all areas where DRx is expressed (Fig. 3F, G, yellow arrowheads), only in the region of the protocerebral bridge (PB) more RFP marker expression is seen (Fig. 3F, G, red arrowheads). In living flies the RFP marker expression was seen in the clypeus (Fig. 3H, red arrowhead), a structure where DRx is necessary for its correct development and which is missing in DRx mutants [7].

We previously identified a DRx mutant allele in an EMS-mutagenesis screen for mutants of the 57B region where DRx is located. In this mutant drx^10155^ a C to T transition generates a stop codon resulting in a shortened DRx protein of only 233 amino acids compared with the 902 amino acids wild-type protein [6]. This shortened protein is also missing the DNA binding domain of DRx, the homeodomain (Fig. S1). The drx^10155^ allele has already been used to analyse the mushroom body phenotype of DRx [24] and is only mentioned herein for completeness.

### Analysis of DRx enhancers

**Fig. 4:**
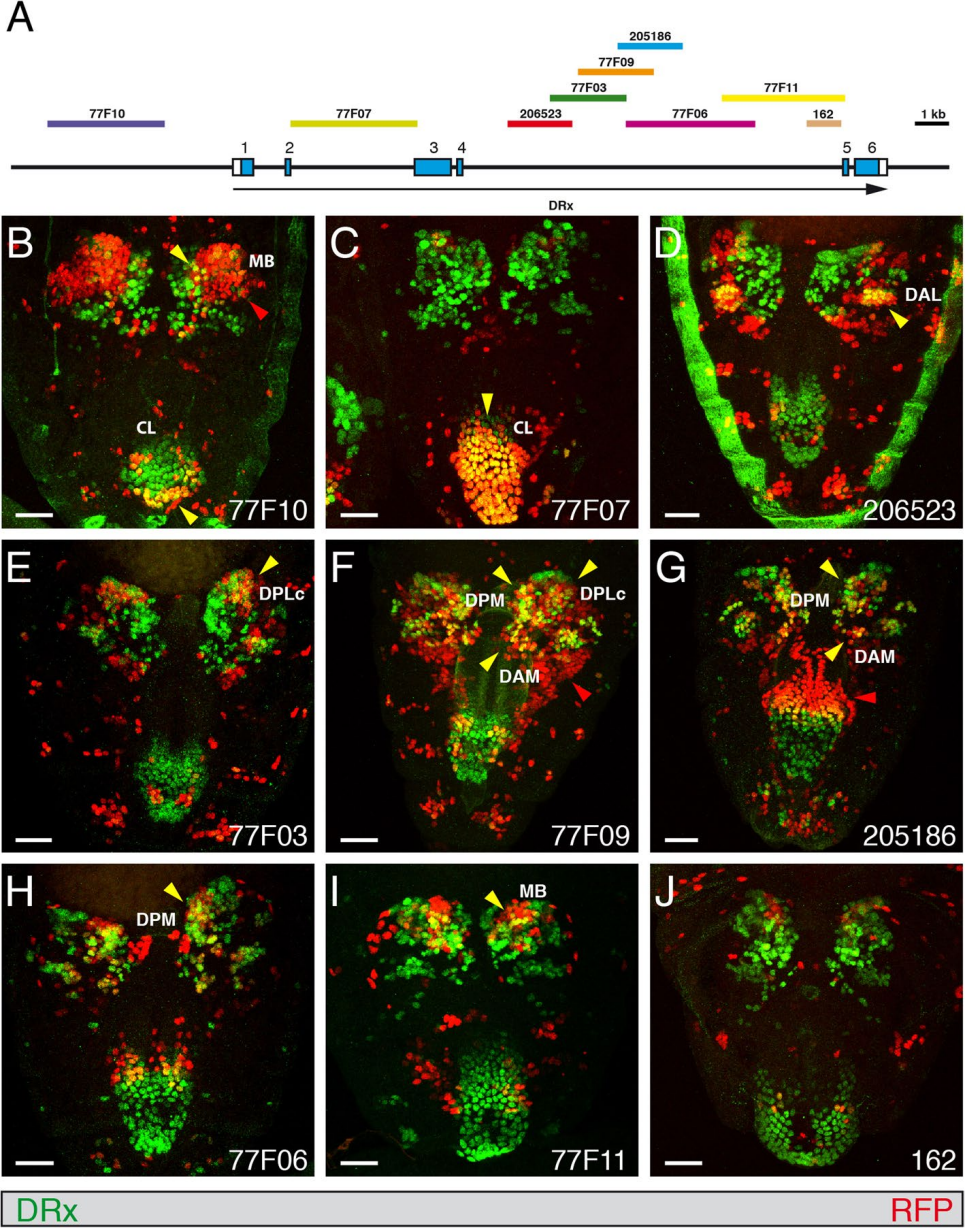
Expression of DRx enhancer-Gal4 strains in the embryo. **A** The genomic organization of the DRx locus is shown together with the location of fragments from the upstream and intronic regions of the DRx locus used to test enhancer activities in the respective Gal4 strains. **B-J** Dorsal views of the anterior parts of stage 15 *Drosophila* embryos. The anterior ends of the embryos are pointing downward. An anti-DRx antibody was used to visualize the nuclear DRx expression pattern in green, and enhancer-Gal4-driven UAS-H2B-mRFP1 expression was used to visualize the nuclear expression patterns generated by the various enhancers in red. The Gal4 strain numbers are indicated; yellow arrowheads indicate important regions showing coexpression of DRx and the fluorescence marker, and red arrowheads denote regions where only the enhancer expression is visible. Brain domains showing coexpression were labelled according to Fig. 1. For symmetrical expression domains arrowheads are only shown for the right side. Abbreviations: CL, clypeolabrum; DAL, dorsal anterior lateral; DAM, dorsal anterior medial; DPLc, dorsal central lateral; DPM, dorsal posterior medial; MB, mushroom body. (Scale bars: 25 μm)

To analyse all putative DRx enhancers we used all available Gal4 strains and one strain we generated with defined fragments from the DRx locus driving Gal4 expression. Six Gal4 fly lines from Janelia Research Campus [47] and two Gal4 strains from the Vienna Tiles Gal4 library (VDRC) [50] covering the upstream region and the two largest introns of the DRx locus were analysed in different developmental stages (Fig. 4A). The Gal4 strains were initially crossed with a UAS-H2B-mRFP1 strain [70] to visualize the pattern of putative enhancers as nuclear stainings in the respective areas compared with the nuclear expression of DRx using an anti-DRx antibody. Later, we recombined the enhancer Gal4 strains carrying insertions on the third chromosome with the UAS-H2B-mRFP1 marker, which was also located on the third chromosome, and balanced the resulting strain. In this way we obtained strains expressing the RFP marker in all animals, facilitating further analyses. When performing such an analysis, one must always consider a potential temporal delay of reporter expression compared with the DRx expression. Conversely, reporter expression might be more stable and therefore might persist for a longer time period. First, the expression of all Gal4 strains was analysed in stage 15 embryos. Strain 77F10 covering a part of the upstream region showed prominent expression of the reporter in the brain (Fig. 4B, red arrowhead) which only partially overlapped with DRx (Fig. 4B, yellow arrowhead). The cells were mushroom body progenitor cells that showed a prolonged expression of the reporter gene with respect to the DRx expression [24]. In addition, some cells in the clypeolabrum (CL) also showed coexpression (Fig. 4B, yellow arrowhead). Strain 77F07, with a fragment from the second intron, showed no expression in the brain, but colocalization in most cells of the clypeolabrum (CL) (Fig. 4C, yellow arrowhead), another expression domain of DRx [6, 7]. In strain 206523, coexpression was observed in a lateral position of the brain in domain DAL (Fig. 4D, yellow arrowhead). A more central coexpression was visible in strain 77F03 in domain DPLc (Fig. 4E, yellow arrowhead). In strain 77F09, very prominent coexpression was observed in the medial region of the brain in domains DAM and DPM and again in domain DPLc (Fig. 4F, yellow arrowheads). An additional domain showing only reporter expression was also visible (Fig. 4F, red arrowhead). Coexpression in some cells in the medial region in domains DAM and DPM was detected in strain 205186 (Fig. 4G, yellow arrowheads), but a larger expression domain was apparent for the reporter alone in the dorsal fold of the embryo (Fig. 4G, red arrowhead). Strain 77F06 also showed coexpression in some cells of the medial region in domain DPM (Fig. 4H, yellow arrowhead), and strain 77F11 showed greater coexpression in the central region in domain MB, but much less compared to strain 77F10 (Fig. 4I, yellow arrowhead). In contrast, strain 162 showed no coexpression (Fig. 4J). In summary, distinct regulatory elements for the expression in the mushroom body progenitors, the clypeolabrum and lateral, central and medial brain regions could be identified.

**Fig. 5:**
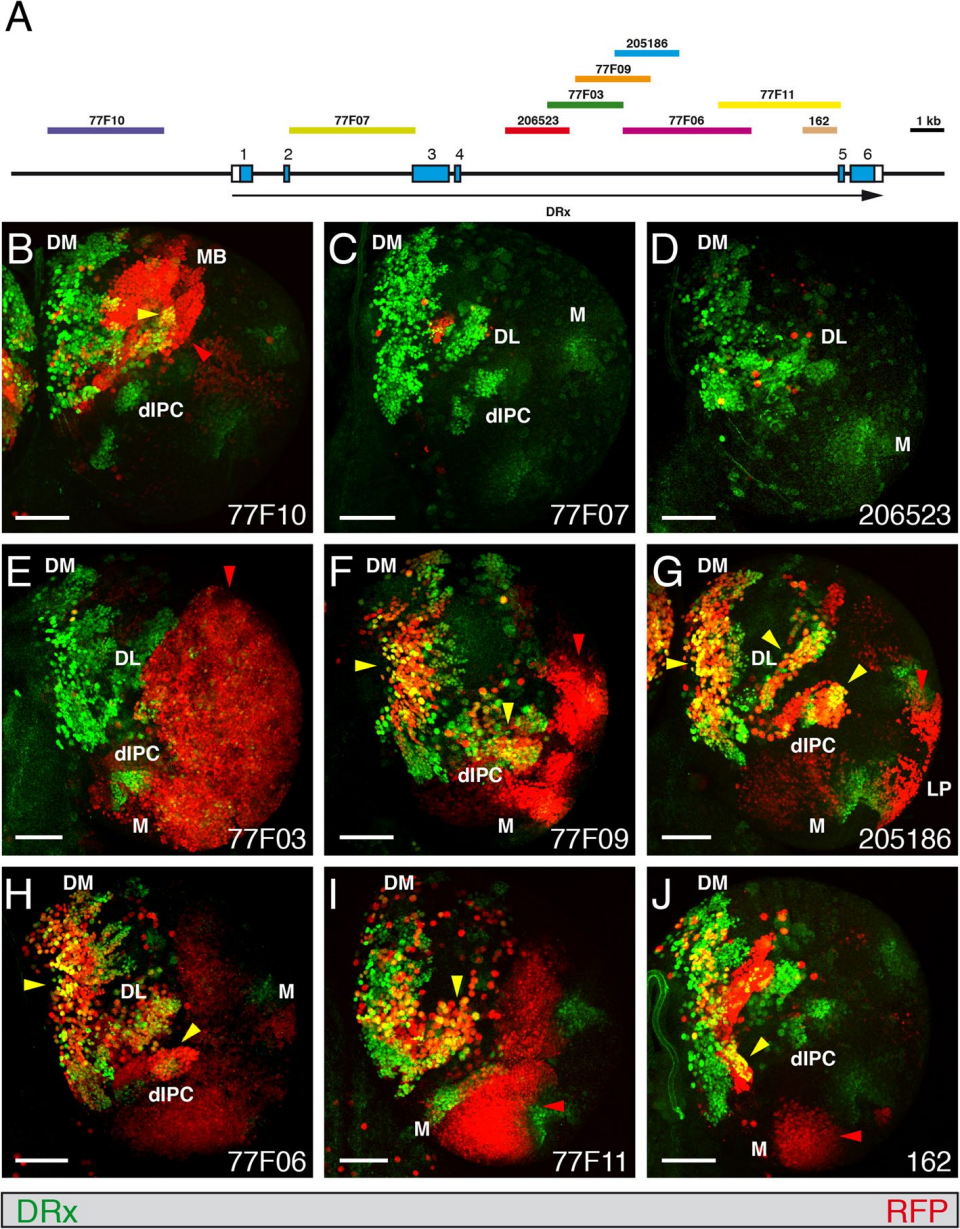
Expression of enhancer-Gal4 strains in the larval brain. **A** The genomic organization of the DRx locus is shown together with the location of fragments from the upstream and intronic regions of the DRx locus used to test enhancer activities in the respective Gal4 strains. **B-J** Views of right hemispheres of *Drosophila* L3 larval brains. An anti-DRx antibody was used to visualize the nuclear DRx expression pattern in green, and enhancer-Gal4-driven UAS-H2B-mRFP1 expression was used to visualize the patterns generated by the various enhancers in red. The Gal4 strain numbers are indicated; yellow arrowheads indicate important regions showing coexpression of DRx and the fluorescence marker, and red arrowheads show regions where only the enhancer expression is visible. dIPC, dorsal inner proliferation centre; DL, dorsolateral lineages; DM, dorsomedial lineages; LP, lobula plate; MB, mushroom body; M, medulla. (Scale bars: 50 μm)

Next, we analysed the expression of the same strains in L3 larval brains. Here, expression of the upstream enhancer 77F10 showed overlap with DRx in some areas of the mushroom body, as in the embryo (Fig. 5B, yellow arrowhead), but again also a larger area where only the reporter was expressed (Fig. 5B, red arrowhead). Strain 77F07, which showed embryonic expression in the clypeolabrum like strain 206523, also demonstrated no expression in the larval brain except for very few cells, similar to strain 206523 (Fig. 5C, D). Strain 77F03 showed very prominent reporter expression in the optic lobe, in the medulla (Fig. 5E, red arrowhead). In strain 77F09, coexpression with DRx was observed in the dorsomedial region and the dorsal inner proliferation centre (Fig. 5F, yellow arrowheads), and only reporter expression was observed in the medulla (Fig. 5F, red arrowhead). The same coexpression was observed in strain 205186, with one additional area of coexpression identified in the dorsolateral region (Fig. 5G, yellow arrowheads). Expression of the reporter was also visible in the lobula plate (Fig. 5G, red arrowhead). 77F06 showed coexpression in the dorsomedial region, though slightly less than 77F09 and 205186 (Fig. 5H, yellow arrowhead), and in the dorsal inner proliferation centre (Fig. 5H, yellow arrowhead). Strain 77F11 showed some coexpression in the region close to the dorsomedial lineages (Fig. 5I, yellow arrowhead) and strong reporter expression in the posterior ventral part of the medulla (Fig. 5I, red arrowhead). Strain 162 showed a pattern similar to 77F11 (Fig. 5J, yellow arrowhead), but it was much weaker in the posterior ventral medulla (Fig. 5J, red arrowhead). The expression of DRx was more apparent in deeper sections of the lobula; here staining was visible in strains 205186 and 77F06 (Fig. S2, red arrowheads).

Similar to the embryo, distinct regulatory elements driving expression in the larval brain in the mushroom body, the dorsomedial lineages, the dorsolateral lineages, the dorsal inner proliferation centre, medulla and lobula plate could also be identified.

**Fig. 6:**
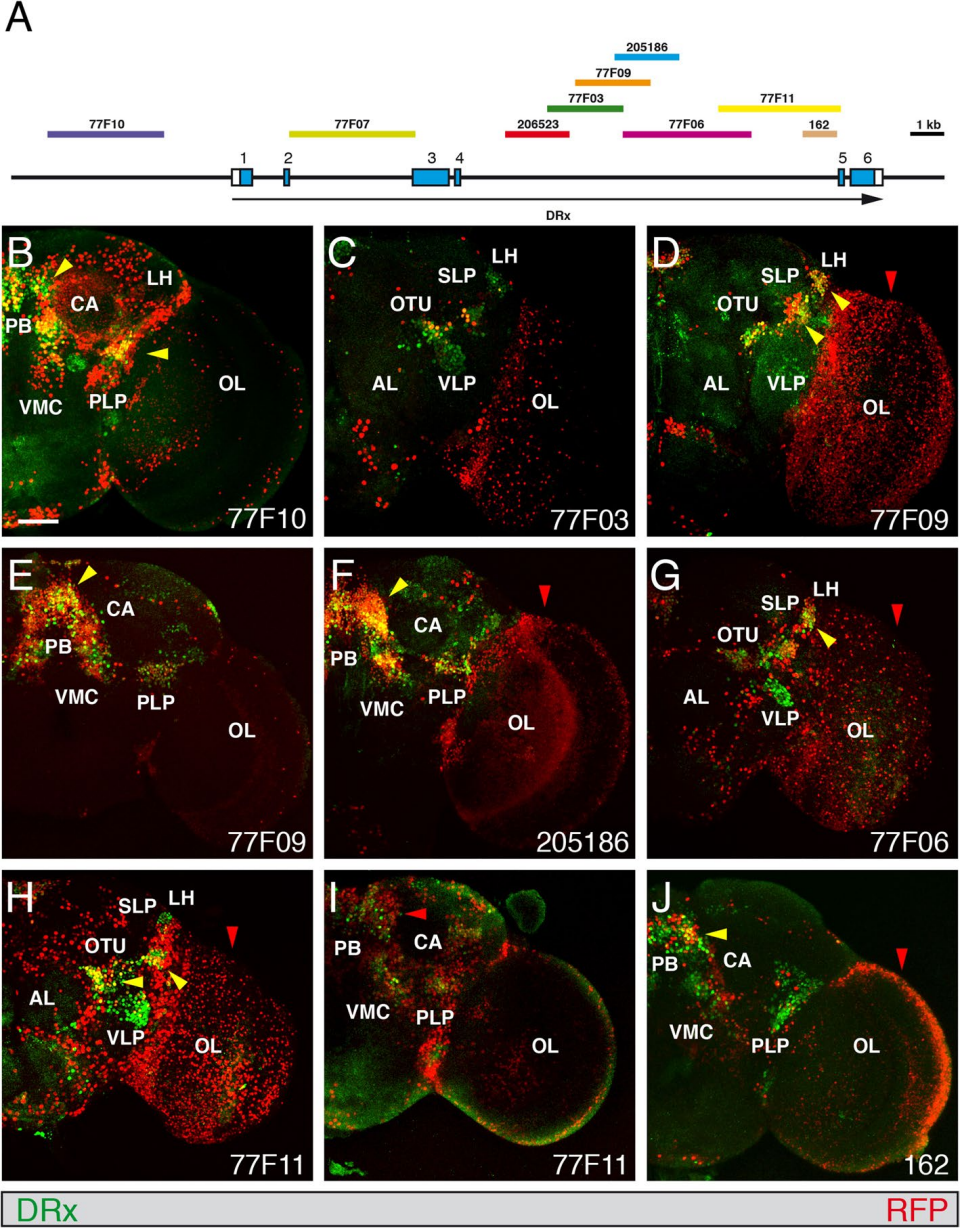
Expression of enhancer-Gal4 strains in the adult brain. **A** The genomic organization of the DRx locus is shown together with the location of fragments from the upstream and intronic regions of the DRx locus used to test enhancer activities in the respective Gal4 strains. **B-J** Views of the right side of *Drosophila* adult brains. An anti-DRx antibody was used to visualize the nuclear DRx expression pattern in green, and enhancer-Gal4 driven UAS-H2B-mRFP1 expression was used to visualize the patterns generated by the various enhancers in red. The Gal4 strain numbers are indicated, yellow arrowheads indicate important regions showing coexpression of DRx and the fluorescence marker, and red arrowheads denote regions where only the enhancer expression is visible. Abbreviations: AL, antennal lobe; CA, calyx (mushroom bodies); LH, lateral horn; OL, optic lobe; OTU, optic tubercle; PB, protocerebral bridge; PLP, posterior-lateral protocerebrum; SLP, superior-lateral protocerebrum; SMP, superior-medial protocerebrum; SOG, subesophageal ganglion; VL, vertical lobe (mushroom bodies); VMC, ventro-medial cerebrum; VLP, ventro-lateral protocerebrum. (Scale bar: 50 μm)

In the adult brain, the upstream enhancer 77F10 showed coexpression of DRx and the nuclear RFP marker in the protocerebral bridge (PB) region and close to the posterior-lateral protocerebrum (PLP) (Fig. 6B, yellow arrowheads), whereas enhancer strains 77F07 and 206523 showed no coexpression. Strain 77F03 showed only a few cells with coexpression in an anterior section of the brain. In strain 77F09 coexpression was detected in the domains close to the superior-lateral protocerebrum (SLP) and dorsal of the ventro-lateral protocerebrum (VLP) (Fig. 6D, yellow arrowheads) and in the protocerebral bridge (PB) region (Fig. 6E, yellow arrowhead). This coexpression in the protocerebral bridge (PB) region was also seen in enhancer strain 205186 (Fig. 6F, yellow arrowhead). Enhancer strain 77F06 showed again coexpression only in a few cells close to the lateral horn (LH) (Fig. 6G, yellow arrowhead). Coexpression of DRx and RFP was seen in the enhancer strain 77F11 in the domains close to optic tubercle (OTU) and a lateral domain close to the optic lobe (OL) (Fig. 6H, yellow arrowheads). In the protocerebral bridge (PB) region a few cells showed coexpression, but there is more RFP marker expression compared to DRx expression (Fig. 6I, red arrowhead). Enhancer strain 162 showed again coexpression only in a few cells (Fig. 6J, yellow arrowheads). Additionally, the enhancer strains 77F09, 205186, 77F06, 77F11 and 162 showed RFP marker expression in the adult optic lobe (Fig. 6D, F, G, H, J, red arrowheads). In adult flies of strain 77F07 we again observed RFP expression in the clypeus, as previously shown for the DRx^Gal4^ enhancer trap strain (Fig. 3G).

**Fig. 7:**
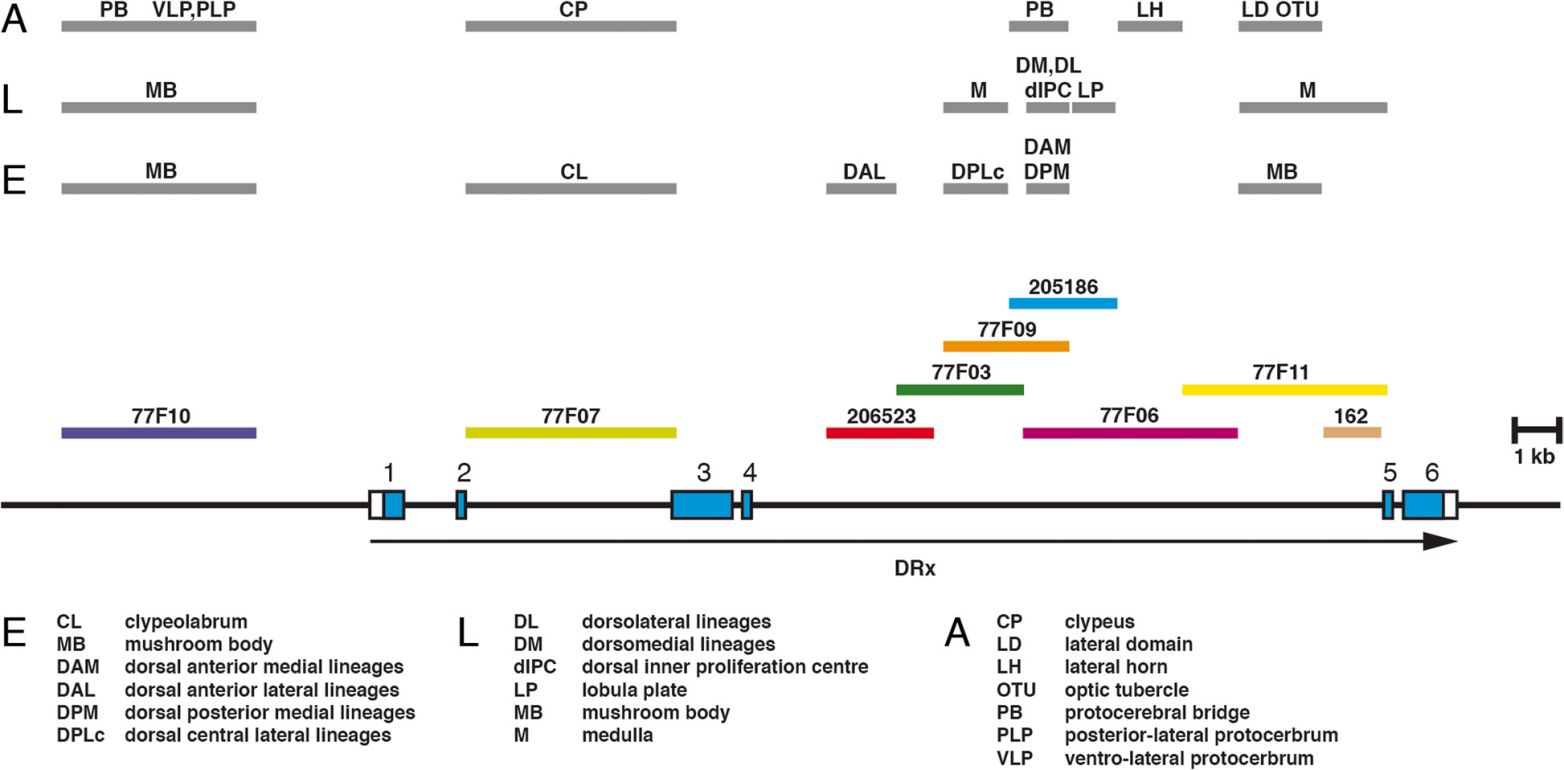
Location of putative enhancers of the the DRx gene driving expression at different developmental stages. The genomic organization of the DRx locus is shown together with the location of fragments from the upstream and intronic regions of the DRx locus used to test enhancer activities in the respective Gal4 strains. Above the genomic organization are the locations of the different putative DRx enhancers indicated as grey boxes at different developmental stages in the embryonic brain (E), larval brain (L) and adult brain (A). For the DRx enhancers in the brain the specific expression domains are indicated according to Figs. 4, 5 and 6. Abbreviations: CL, clypeolabrum; CP, clypeus; DAL, dorsal anterior lateral; DAM, dorsal anterior medial; dIPC, dorsal inner proliferation centre; DL, dorsolateral lineages; DM, dorsomedial lineages; DPLc, dorsal central lateral; DPM, dorsal posterior medial; LD, lateral domain; LH, lateral horn; LP, lobula plate; M, medulla; MB, mushroom body; OTU, optic tubercle; PB, protocerebral bridge; PLP, posterior-lateral protocerebrum; VLP, ventro-lateral protocerebrum

A schematic summary of the location of all the different putative enhancers of the DRx gene is shown in Fig. 7. In the embryo we identified enhancers driving expression in the clypeolabrum, mushroom body progenitors and in the DAL, DAM, DPLc and DPM regions of the embryonic brain. For the few cells in the BLD domain, we could not clearly identify an enhancer. Identified enhancers in the larval brain drive expression in the mushroom bodies, dorsomedial and dorsolateral lineages, dorsal inner proliferation centre, medulla and lobula plate. In the adult brain we identified regions responsible for DRx expression in the LD, LH, OTU, PLP/VLP and PB regions and one for expression in the clypeus. Some enhancers drove expression in structures that are continuously developing. For example, enhancer 77F10 is active in the mushroom body progenitors in the embryo and also in the larval mushroom body. Another example is the enhancer of dorsomedial expression in the larval brain, which was already active in the medial region of the embryo where the dorsomedial lineages originate from. Enhancer 77F07 drove expression in the clypeolabrum in the embryo and then in the clypeus of the adult. This enhancer is most likely also active at the larval stage, but we did not further examine this possibility.

### Generation of enhancer deletions by gene targeting

**Fig. 8:**
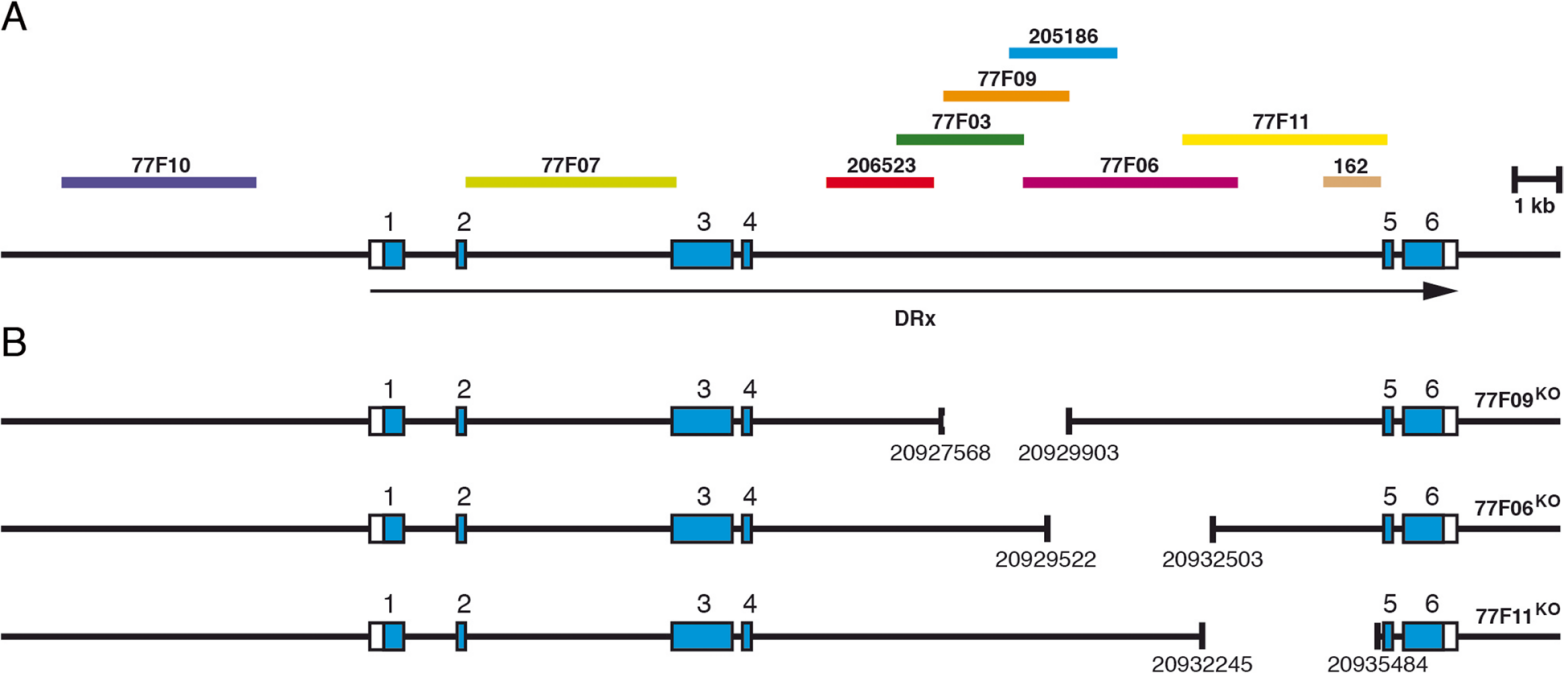
DRx enhancer gene targeting strains. **A** The genomic organization of the DRx locus is shown together with the location of fragments from the upstream and intronic regions of the DRx locus used to test enhancer activities in the respective Gal4 strains. **B** The individual enhancer deletion strains 77F09^KO^, 77F06^KO^ and 77F11^KO^ with respective deleted regions are indicated. Deletion breakpoint positions are indicated according to the sequences from Flybase (FB2021_04)

Analysis of all putative enhancers in the DRx locus showed that 77F09, 77F06 and 77F11 accounted for the most prominent expression pattern of DRx and drove expression in the major areas of DRx expression in the embryonic brain and the dorsomedial and dorsolateral lineages and the lobula plate in the larval brain. To functionally examine some DRx enhancers, constructs for a gene targeting experiment were generated to delete individual enhancers by gene targeting via homologous recombination (Fig. 8A, B). For the enhancer construct 77F09, which has a length of 2.3 kb, we planned a deletion of the complete region (77F09^KO^). In the case of the enhancer constructs 77F06 and 77F11, both of which are 3.8 kb long and have an overlapping region of 1.0 kb, we generated targeting constructs with smaller deletion regions to avoid overlaps that were too large here (3.0 kb for 77F06^KO^ and 3.2 kb for 77F11^KO^ with an overlap of 0.26 kb). This strategy also allowed us to assign putative effects more specifically to certain regions. Similar to the DRx gene targeting construct, we again PCR amplified 2.7 kb homology arms, cloned them into the pTV^cherry^ vector, made transgenic flies and generated targeting flies through the appropriate fly crosses. In the case of 77F11^KO^ we screened 38,135 flies and identified 21 red-eyed flies (1/1816), for 77F09^KO^, we screened 47,318 flies and identified 8 red-eyed flies (1/5914) and for 77F06^KO^, we screened 22,517 flies and recovered 6 red-eyed flies (1/3506). In all cases, the white gene was removed, and the final strains 77F06^KO^, 77F09^KO^ and 77F11^KO^ were molecularly analysed by PCR and sequencing of the deletion breakpoints. The three strains were balanced and further evaluated.

### Functional analysis of DRx enhancer deletion strains

**Fig. 9:**
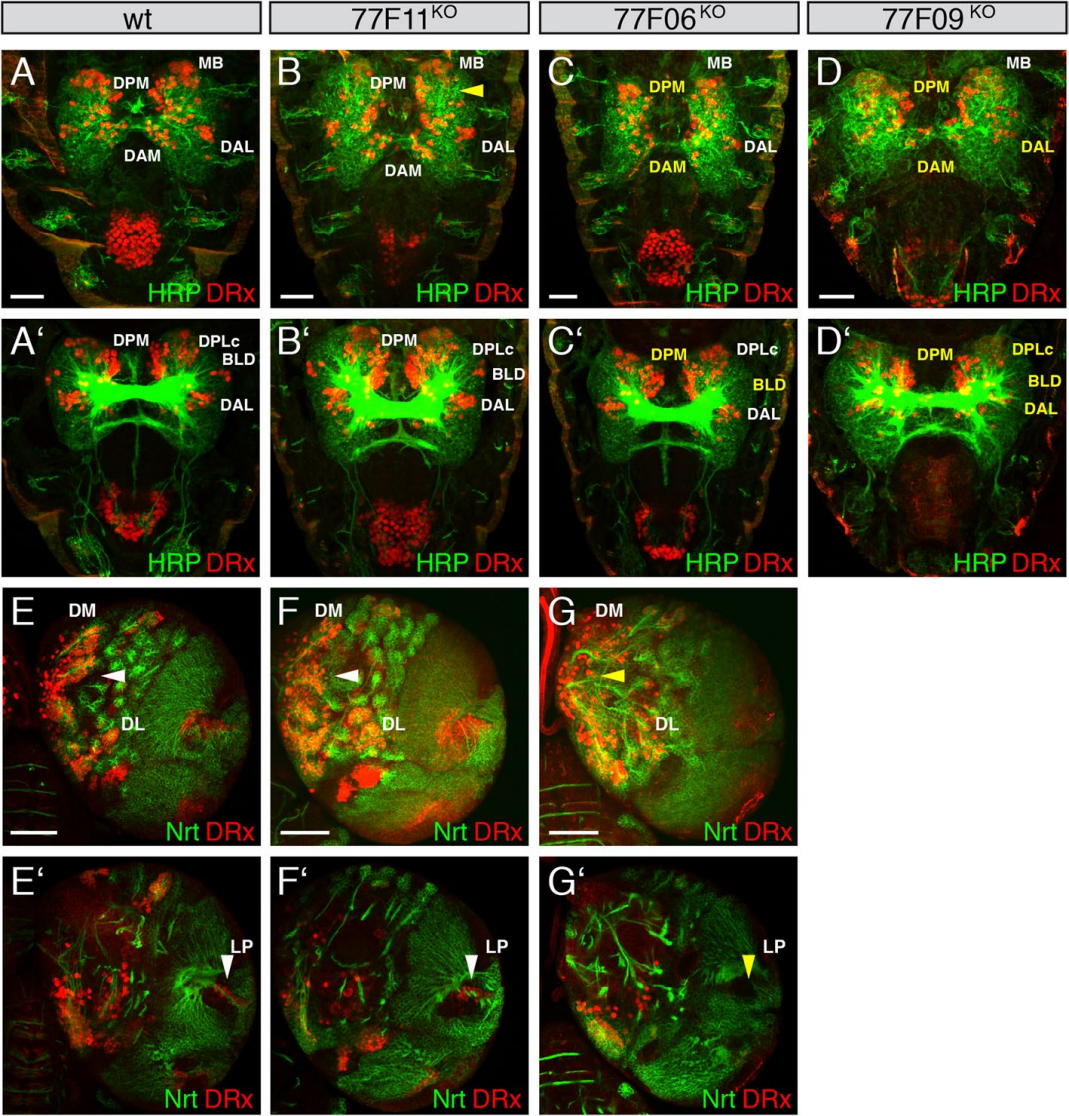
Analysis of DRx enhancer gene targeting strains. **A-D′** Laser confocal images of the anterior parts of stage 16 *Drosophila* embryos stained using HRP (green) and DRx (red). The anterior ends of the embryos are pointing to the bottom. For each embryo two different focal planes are shown (**A, A‘**to **D, D‘**). Expression domains are indicated according to the wild-type (**A, A‘**). Expression domains with reduced or missing DRx expression in the enhancer gene targeting strains are indicated in yellow. **E-G‘**Laser confocal images of right hemispheres from third instar larval brains. Again two different focal planes are shown (**E, E‘**to **G, G‘**). Staining was performed using Nrt (BP106) (green) and DRx (red). (A, A’) DRx expression in a wild-type embryo is shown as a reference. The expression domains BLD, DAL, DAM, DPLc, DPM, MB reflect those in Fig.1. **B, B′** In 77F11^KO^ embryos the DRx expression pattern was similar to that in wild-type embryos. A slight reduction was observed in the central region (yellow arrowhead). **C, C′** 77F06^KO^ embryos show less DRx expression in domains DAM and DPM, and expression in domain BLD is missing. (**D, D′**) The 77F09^KO^ embryos show an altered DRx expression in all domains except the mushroom body domain. Abbreviations: BLD, basal lateral dorsal; DAL, dorsal anterior lateral; DAM, dorsal anterior medial; DPLc, dorsal central lateral; DPM, dorsal posterior medial; MB, mushroom body. **E, E’**) In the wild-type larval brain, prominent expression of DRx can be observed in the DM and DL lineages (white arrowhead), and weaker expression is seen in the lobula plate (white arrowhead). **F, F′** Again, no obvious alterations of the DRx expression pattern were visible compared with the wild-type. **G, G’** In strain 77F06^KO^, the DM lineages looked disorganized and less DRx staining was visible, especially in the upper lineage areas (yellow arrowhead). The DRx expression in the lobula plate was completely gone (yellow arrowhead). Abbreviations: DL, dorsolateral lineages; DM, dorsomedial lineages; LP, lobula plate. (Scale bars: 50 μm)

First, we analysed the lethality of all three DRx enhancer gene targeting strains. Strains 77F06^KO^ and 77F11^KO^ were not lethal, and homozygous adult flies developed. In contrast, strain 77F09^KO^ showed embryonic lethality, which was unexpected since deletion of a regulatory element should not result in an earlier lethality than a null mutation of the gene. Therefore we performed a complementation analysis with a Df(2R)Exel7166 deficiency to uncover part of the 57B region including the complete DRx locus and neighbouring genes. Since the strain 77F09^KO^ showed complementation with this deficiency, the reason for the lethality was determined to be a mutation in a gene outside the DRx locus, and we could only analyse embryos of this strain. In our analysis, we performed DRx staining of embryonic and larval brains combined with general markers and expected a loss of DRx expression in distinct areas that might alter specific brain structures. For staining of stage 16 embryonic brains, HRP was used as a general marker together with DRx and two different focal planes per embryo were shown, a more dorsal one (Fig. 9A-D) and a more ventral at the level of the brain commissure (Fig. 9A’-D’). In the wild-type embryo, DRx expression was visible in specific expression domains that were labelled according to Fig. 1 (Fig. 9A, A’, BLD, DAL, DAM, DPLc, DPM, MB). In strain 77F11^KO^ the DRx pattern in the embryonic brain was very similar to that in the wild-type brain (Fig. 9B, B’). A slight reduction of expression was observed in a central region close to the mushroom body (Fig. 9B, yellow arrowhead). In contrast to strain 77F11^KO^, strain 77F06^KO^ showed a reduction of DRx expression in domain DAM, which was closely associated with the brain commissure, and domain DPM located medially; it also showed a loss of DRx expression in domain BLD located laterally (Fig. 9C, C’). Almost all DRx expression domains were affected in strain 77F09^KO^, wherein no expression was found in domain BLD, and the expression was more or less reduced in all other domains except domain MB (Fig. 9D, D’). We next analysed larval brains of strains 77F11^KO^ and 77F06^KO^, again using different focal planes focusing on the region of the type II DM and DL lineages (Fig. 9E-G) and the lobula complex (Fig. 9E’-G’). The general marker Nrt was used in combination with DRx. In the wild-type larval brain, prominent DRx expression was observed in the DM and DL regions (Fig. 9E, white arrowhead) and in the lobula plate (Fig. 9E’, white arrowhead). DRx expression in strain 77F11^KO^ was comparable to that in the wild-type (Fig. 9F, white arrowhead, Fig. 9F’, white arrowhead). A different effect was observed in strain 77F06^KO^, fewer DRx-positive cells were visible in the DM and DL regions and the lineages appeared disorganized (Fig. 9G, yellow arrowhead). In the lobula no expression of DRx was detectable (Fig. 9G’, yellow arrowhead). In summary, strains 77F06^KO^ and 77F09^KO^ showed similar effects in the embryonic brain. Since the deletions in both strains are slightly overlapped, this effect might have been due to the overlapping region. Larval brains of strain 77F06^KO^ showed a clear loss of DRx expression in the lobula and a reduction of DRx expression in the DM and DL regions.

## Discussion

In this study, we analysed the expression and regulation of the transcription factor DRx during brain development. DRx is expressed in the embryonic, larval and adult brain [7]. In the embryonic brain, expression occurs in 8–10 neuroblasts per hemisphere and in many other cells, including the mushroom body neuroblasts and their progeny [24]. We continued to examine this expression of DRx in postembryonic stages and showed that DRx was expressed in the larval brain in the DM lineages and the lobula complex and in the adults in the central complex. The DM lineages are of particular interest because they belong to the largest lineages in the larval brain, with up to 400 cells, and develop later in structures of the adult central complex [21].

The neuroblasts generating these lineages are already present in the embryonic brain in three clusters: an anterior dorsomedial (ADM) cluster of three neuroblasts, a posterior dorsomedial (PDM) cluster of three neuroblasts and a dorsolateral (DL) cluster of two neuroblasts [17, 18]. ADM cluster neuroblasts will give rise to the DM1–3 lineages, PDM cluster neuroblasts give rise to the DM4–6 lineages and the DL cluster neuroblasts give rise to the DL lineages. Embryonic type II neuroblasts express a series of some known transcription factors, including DRx, Hbn and Otp [39]. DRx and Hbn are only expressed in the PDM and DL clusters, which are missing when all three genes are deleted, whereas the ADM cluster is still present [39]. Even if DRx is not expressed in the ADM cluster, it is expressed in all type II lineages in the larval brain, there in GMCs and neurons in part of the lineages. It is not known when DRx stops being expressed in the type II neuroblasts during development or if it is ever expressed in DM1–3 neuroblasts before the third larval stage. Such a switch of expression in cell types of a defined lineage during development is seen not only for DRx, but also for Hbn, which is no longer expressed in larval type II neuroblasts (Hildebrandt et al., 2021, in preparation) compared with the embryo [39].

In this study we have analysed nine enhancer candidate fragments and identified five enhancer regions that drove marker expression in the embryo, four in the larval brain and three in the adult brain. All of these enhancers could be assigned to DRx expression. Another DRx enhancer analysis using lacZ as a reporter gene was recently published [51] that focused on the DRx expression in the posterior outer proliferation centre and revealed two regions in the largest intron of DRx. These two regions perfectly matched the regions we also identified using our Gal4 constructs, namely the overlapping region of 77F03 and 77F09 and the region located in 77F11. The enhancer fragments we analysed were located upstream and in the two larger introns of DRx. Some enhancers drove expression of DRx during the establishment of specific structures starting in the embryo up to the adult stage. DRx is expressed in the mushroom body and is necessary for cell growth, proliferation and survival of mushroom body neuroblasts [24]. Here, we showed that enhancer 77F10 was responsible for this effect. Another example was enhancer 77F07, which showed expression in the clypeolabrum in the embryo and in the adult clypeus, a structure that is missing in DRx mutants [7]. In a larger analysis of 7705 tested fragments, 46% of these fragments were active in the embryo and were located upstream (30%), downstream (22%) or in introns (36%), and the rest were located further away (12%) [50]. This result is in good agreement with our findings; we identified most enhancers in the two larger introns and one in the upstream region. We did not analyse the region downstream of DRx since only regulatory elements of the neighbouring actin 57B gene were located there (data not shown). We cannot rule out the possibility that enhancers located further away may contribute to accurate DRx expression, but since we identified the enhancers for the most prominent expression patterns of DRx, this may not be the case. We identified two enhancer regions in the embryo and two enhancers driving expression in the medulla of the larval brain, which might be due to redundant enhancers, called shadow enhancers, providing robustness to regulatory networks [71, 72]. This is not such a rare event since a systematic analysis showed that 64% of the loci examined had shadow enhancers, 70% of which had more than one [72].

To generate a DRx strain with reintegration of Gal4 into the DRx locus and further analyse of three DRx enhancers we used the gene targeting method. To perform our gene targeting experiments, we basically followed the experimental design suggested by Baena-Lopez et al. [69]. They generated a new vector for accelerated homologous recombination and subsequent genome modification in *Drosophila* [69]. Using this strategy not only deletions but also the reintegration of other pieces of DNA in the locus, such as cDNAs, Gal4, Gal80 or fluorescent markers, can be made. As a proof of principle, the researchers inactivated genes such as *hedgehog* (*hh*), *wingless* (*wg*) and *rhogap 102A* by deletion of part of the first exon starting a few base pairs upstream of the ATG and downstream sequences, including the donor splice sites [69]. We constructed our DRx construct in the same manner and verified the correct targeting event molecularly. Nevertheless, our DRx targeting fly strain was not lethal, as expected, and showed DRx expression. The only available explanation for this phenomenon was the use of a downstream ATG leading to a shorter N-terminal truncated DRx protein that was still functional. In addition to the DRx cDNA we isolated [6], another cDNA is also available (RE39020, flybase), in which a different ATG in a different reading frame is present in exon 1; however, since this ATG is downstream of the one we considered, it would be also eliminated by the targeting event. Since there is no ATG in exon 2 and the next ATG is in exon 3, the use of this ATG would eliminate the first 193 amino acids of DRx including the octapeptide. The Octapeptide is a repressor domain, whose deletion in mouse Pax-2 results in increased transactivation by Pax proteins [73], therefore a similar effect might also apply here.

The efficiency of gene targeting mainly depends on three parameters: the length of the homology arms, the size of the region which has to be deleted and the chromosomal integration of the targeting construct used for the targeting via homologous recombination. We generated our first targeting construct with the pTV^cherry^ vector for the gene *homeobrain* with 4.0 kb homology arms (unpublished results) and reduced the length herein to 2.7 kb, retaining comparable efficiency (1/600–1/700) in the case of the DRx targeting constructs, where the generated deletion was also rather small. If the deleted region was larger, then the efficiency dropped to 1/1816–1/5918, similar to the enhancer deletion constructs. This variation could also depend on the initial integration sites of the constructs, since we observed efficiency differences of up to threefold for the same donor construct depending on the chromosomal integration site. In general, our targeting efficiencies were similar to those reported by Baena-Lopez et al. [69]. In the case of the 77F09^KO^ strain, we finally determined that the strain was embryonic lethal, which was unexpected. This lethality may have been due to the integration sites of the targeting donor constructs. If a donor construct integration had led to some lethality, then flippase-induced recombination of the construct out of the chromosome would leave some P-element sequences at that position, and lethality might persist. During our gene targeting experiments in the lab, we encountered such a case and followed the integration of the donor construct molecularly by cloning sequences adjacent to the donor construct integration; indeed the construct was integrated into a gene and inactivated that gene. Therefore, it would be advisable to use only construct donor strains with nonlethal integrations and in the ideal situation located on a different chromosome relative to the targeting locus so that resident P-element sequences might be lost over time in the final targeting strain. The advantage of specific enhancer deletions using gene targeting versus RNAi downregulation using defined Gal4 strains is that the deletions represent new tissue-specific DRx alleles that should have a defined and reproducible phenotype. Downregulation by RNAi, on the other hand, might not be as effective and could also have a temporal delay due to RNAi activation using the UAS/Gal4 system.

Our analysis of the enhancer gene targeting strains showed that the 77F11 enhancer deletion showed no obvious alterations in DRx expression, and subtle changes may have occurred that can only be detected by a more detailed analysis using specific markers. Another possibility might be a previously mentioned enhancer redundancy. In contrast, deletion of the enhancer 77F06 had clear effects in the embryonic brain and the larval brain, herein resulting in a disorganization of the DM lineages and a loss of DRx expression in the lobula. The 77F09 enhancer deletion appeared similar to the 77F06 deletion in the embryo, with even some more DRx-expressing cells missing.

A more detailed analysis of these enhancers at the level of expression and function using more specific markers will hopefully provide better insights into the function of individual enhancers and how they cooperate together to regulate DRx expression in time and space. Since in enhancer deletions by gene targeting attP sites have been introduced into the locus instead of the deleted enhancer, as in our experimental design [69], all types of rescue experiments could also be performed. It is possible to reintegrate shorter enhancer fragments, homologous fragments from other *Drosophila* species or modified enhancer fragments that might have deletions or binding site mutations for identified transcription factors. In the case of the enhancer 77F06 this could be the transcription factor Optix which was shown to bind in a region of the 77F06 enhancer [25, 51].

## Conclusions

Using a detailed gene expression analysis, we showed that the *Drosophila* homeodomain transcription factor DRx had a very dynamic expression pattern during development. It was expressed in neural stem cells or neurons depending on the developmental stage and various lineages, and it was therefore determined to be an important factor for brain development. This DRx expression was regulated by several well-defined enhancers in the upstream and intronic regions of the gene. The generation of three enhancer deletions using gene targeting is an initial step towards a deeper functional analysis of these enhancers in the future.

## Methods

### Fly strains

The following fly strains were used: yw67c3; UAS-H2B-mRFP1, UAS-mCDC8::GFP [70] and ubiquitin-Gal4[3xP3-GFP] [69]. The following stocks were obtained from the Bloomington Drosophila Stock Center (BDSC) and the Vienna Drosophila Research center (VDRC):

- y [1] w[67c23]; sna[Sco]/CyO, P{w[+mC] = Crew}DH1 (BL 1092);
- y [1] w[*]; Pin[Yt]/CyO; P{w[+mC] = UAS-mCD8::GFP.L}LL6 (BL 5130),
- w[1118] (BL 5905)
- y [1] w[1118]; P{ry[+t7.2] = 70FLP}23 P{v[+t1.8] = 70I-SceI}4A/TM3, Sb [1] Ser [1] (BL 6935)
- w[1118]; Df(2R)Exel7166/CyO (Bl 7998),
- y [1] w[1118]; PBac{y[+]-attP-3B}VK00033 (BL 9750);
- y [1] w[*] P{y[+t7.7] = nos-phiC31\int.NLS}X; sna[Sco]/CyO (BL 34770);
- w[1118]; P{y[+t7.7] w[+mC] = GMR77F03-GAL4}attP2 (BL 39972);
- w[1118]; P{y[+t7.7] w[+mC] = GMR77F07-GAL4}attP2 (BL 39973);
- w[1118]; P{y[+t7.7] w[+mC] = GMR77F06-GAL4}attP2 (BL 46985);
- w[1118]; P{y[+t7.7] w[+mC] = GMR77F09-GAL4}attP2 (BL 46986);
- w[1118]; P{y[+t7.7] w[+mC] = GMR77F11-GAL4}attP2 (BL 46987);
- P{VT020018-GAL4}attP2 (VDRC 205186);
- P{VT020016-GAL4}attP2 (VDRC 206523).

### Generation of a DRx gene targeting construct

A DRx donor gene targeting construct was made in the vector pTV^cherry^ according to Baena-Lopez et al. [69]. The two 2.7 kb homology arms were amplified using Pfu DNA Polymerase (New England Biolabs) and BACR10P11 DNA [74]. Primers RxGT1 (5′-GAATTCGAATGGGAATAAGGAGAGG-3′) and RxGT2 (5′-GGTACCGGGGCAAGAGTACTTAAATCGGC-3′) were used for homology arm 1, and RxGT3 (5′-ACTAGTGACGGCAAATTTCGAGGGTCTAC-3′) and RxGT4 (5′-GGCGCGCCATCTCGTGTAGATGGATCGTCGTG-3′) were used for homology arm 2. All primers contained unique restriction enzyme recognition sites, which were added to their ends (underlined), enabling later cloning in the final vector. After the addition of 3′ adenine overhangs to two PCR products, they were subcloned into the vector pCR 2.1 (Thermo Fisher Scientific, Waltham, Massachusetts, USA) and checked by sequencing. From the correct clones, homology arms were excised with the relevant restriction enzymes and finally cloned into the vector pTV^cherry^ [69]. P-element-mediated transformation into *w*^*1118*^ flies was performed by BestGene (Chino Hills, California, USA). Transformants were balanced, and transformants with integration on the third chromosome were used to generate the final targeting strain. Transformants were crossed with *hs-Flp, hs-SceI* flies (BL 26579), and resulting larvae were heat shocked at 48 h and 72 h after egg laying for 1 h at 37 °C. Two hundred adult female flies with mottled red eyes were crossed with *ubiquitin-Gal4[3xP3-GFP]* males, and the progeny were screened for the presence of red-eyed flies. The transgene *ubiquitin-Gal4[3xP3-GFP]* was removed by selection against GFP expression and the resulting targeting flies were balanced over CyO and molecularly analysed for the correct integration event. To verify this finding, we performed PCRs with primers within the cassette introduced by the recombination events and primers located outside of the homology arms (RxGT5 (5′-CAGACGCACCTGGAGAGTGC-3′), mCherryrev2 (5′-CCTCGTCGTCGTTCAGGTTG-3′) for the upstream region and pTVGal4–1 (5′-CGTTTTTATTGTCAGGGAGTGAGTTTGC-3′), RxGT8 (5′-TCAATCACAAGTGCTTGTTGTTGGCAG-3′) for the downstream region). From one of these strains, DRx^KO^ removal of the white gene was performed by crossing of the DRx-targeting flies to a strain expressing Cre Rekombinase (BL 1092) and selecting for and balancing of white eyed flies among the cross offspring. For the reintegration of Gal4 in the DRx locus the vector RIV^Gal4^ was used [69]. DRx-targeting flies were crossed with PhiC31-expressing flies (BL 34770) and embryos of that cross were injected with RIV^Gal4^ DNA. Red-eyed transformant flies were selected, and the white marker was again removed using the loxP sites to generate the strain DRx^Gal4^.

### Generation of DRx enhancer deletions by gene targeting

DRx donor constructs for the deletion of enhancer regions were generated in the same way as was described for the DRx gene targeting construct using BACR10P11 DNA [74]. In all cases, homology arms of approximately 2.7 kb were PCR-amplified using GT1 and GT2 primers for homology arm 1 and GT3 and GT4 primers for homology arm 2. The following primers were used: 77F06GT1 (5′-GCGGCCGCAGATGGGATTGGGATATACGGAG-3′), 77F06GT2 (5′-GGTACCTGGCTGTTTTCTCAGAGATGCAAGG-3′), 77F06GT3 (5′-ACTAGTCATAATATGCTTATGCCATACGTTGG-3′), and 77F06GT4 (5′-AGATCTTGGCTCTAATTAGAATTATCGCAAC-3′) for construct 77F06GT; 77F09GT1 (5′-GCGGCCGCCCCATATCTTTCTGTGTAGTCTCC-3′), 77F09GT2 (5′-GGTACCAGCCTACTTAAGCATTCAATGG-3′), 77F09GT3 (5′-ACTAGTCAATTATGACTCTGATTTCGGATTGTG-3′), and 77F09GT4 (5′-GGCGCGCCGTTTTCGTACGGCGATAGG-3′) for construct 77F09GT; 77F11GT1 (5′-GCGGCCGCATCTCTGAGAAAACAGCCAGC-3′), 77F11GT2 (5′-GGTACCGTAATAACCCCAATGCGAATTGC-3′), 77F11GT3 (5′-ACTAGTGCTTAACGCCCGACTAACTTAGC-3′), and 77F11GT4 (5′-GGCGCGCCTGTAGCGGGGACGCACAC-3′) for construct 77F11GT. To confirm that the deletions conformed to the prediction, we PCR-amplified the deletion breakpoints (primer sequences are available upon request) and the PCR products were sequenced by Starseq (Mainz, Germany).

### Immunostaining

Embryos were collected, dechorionated with 50% bleach for 2 min, washed with 0.1% NaCl /0.1% Triton X-100 and fixed for 12 min in 3.7% formaldehyde in PEM (100 mM PIPES, 1 mM EGTA, 1 mM MgCl_2_) and heptane. After removal of both phases, embryos were devitelinized in equal volumes of heptane and methanol by 2 min of vigorous shaking and washed three times with methanol. The 3rd instar larvae and adult brains were dissected in 1x phosphate buffered saline (PBS), fixed for 60 min in 2% paraformaldehyde in PBL and washed three times with 1x PBS containing 0.2% Triton X-100 (PBX) and then incubated for 3 × 5 min in methanol. Fixed embryos or larval brains were washed 3 × 5 min and 6 × 30 min in PBX and blocked for 30 min in 5% normal horse serum and 10% PBX in PBS. Incubations with primary antibodies were performed overnight at 4 °C. Samples were washed 3 × 5 min and 6 × 30 min in PBX and blocked for 30 min in 5% normal horse serum and 10% PBX in PBS. After an overnight incubation with secondary antibodies at 4 °C embryos or larvae were washed 3 × 5 min and 6 × 30 min in PBX and mounted in Vectashield (Vector Laboratories). Adult brains were treated the same as larval brains but were incubated with the appropriate antibody two nights each. Images were obtained using an Olympus BX61 microscope (Olympus, Hamburg, Germany) for DIC microscopy, an Olympus SZX12 microscope (Olympus, Hamburg, Germany) for fluorescence images of adult *Drosophila* heads and a Leica TCS SP5 microscope (Leica, Wetzlar, Germany) or a ZEISS LSM 710 microscope (Carl Zeiss AG, Oberkochen, Germany) for laser confocal microscopy. Usually z-stacks of 1 μm were generated and several stacks combined to show the relevant structure or expression domain completely. Images were processed using FIJI and ImageJ (NIH. Md., USA), Adobe Photoshop and Adobe Illustrator (Adobe Systems, San Jose, CA, USA).

Primary antibodies used were rabbit anti-DRx antibody (1:1000) [7], goat FITC-conjugated anti-HRP antibody (1:100) (ICN Biomedical/ Cappel); rabbit anti-Elav antibody (1:30), mouse anti-Repo antibody (1:10), mouse anti-Pros antibody (1:10), mouse anti-Brp (nc82) (1:25) and mouse anti-Nrt (BP106) antibody (1:25) were obtained from the Developmental Studies Hybridoma Bank, Iowa. Secondary antibodies were goat anti-mouse, anti-rabbit and anti-guinea conjugated with Alexa 488, 568 and 647 (1:1000, Molecular Probes, Eugene, Oregon, USA).

## Supplementary Information

**Additional file 1: Figure S1.** Molecular analysis of the DRx^10155^ allele. Nucleotide and amino acid sequences in wild-type (here DRx1 cDNA as an example) and mutant DNA. EMS induced a C to T transition in the coding region of DRx^10155^ leading to the formation of a stop codon. Schematic overview of the wild-type DRx protein with the localisation of the octapeptide (red), the homeodomain (yellow), the Rx domain (green) and the OAR domain (blue) in comparison to the truncated mutant protein of the DRx^10155^ allele.

**Additional file 2: Figure S2.** Expression of enhancer-Gal4 strains in the lobula. (A, B) Views of right hemispheres of *Drosophila* L3 larval brains. An anti-DRx antibody was used to visualize the nuclear DRx expression pattern in green, and enhancer-Gal4 driven UAS-H2B-mRFP1 expression showed the patterns generated by the various enhancers in red. The Gal4 strain numbers are indicated, and the red arrowheads indicate the regions where the enhancer expression is visible. LP, lobula plate. (Scale bar: 50 μm).

## Data Availability

The datasets supporting the conclusions of this article are included within the article. Materials are available from the corresponding author on reasonable request.

## Abbreviations

BDPD: Berkeley Drosophila Genome Project
cDNA: complementary desoxyribonucleic acid
DSHB: Developmental Studies Hybridoma Bank
VDRC: Vienna Drosophila Resource Center
