# Supplementary figures and images for: Functional analysis of enhancer elements regulating the expression of the Drosophila homeodomain transcription factor DRx by gene targeting
Source: Hereditas. 2021 Nov 5;158:42. doi: 10.1186/s41065-021-00210-z (PMC8569992; doi:10.1186/s41065-021-00210-z)

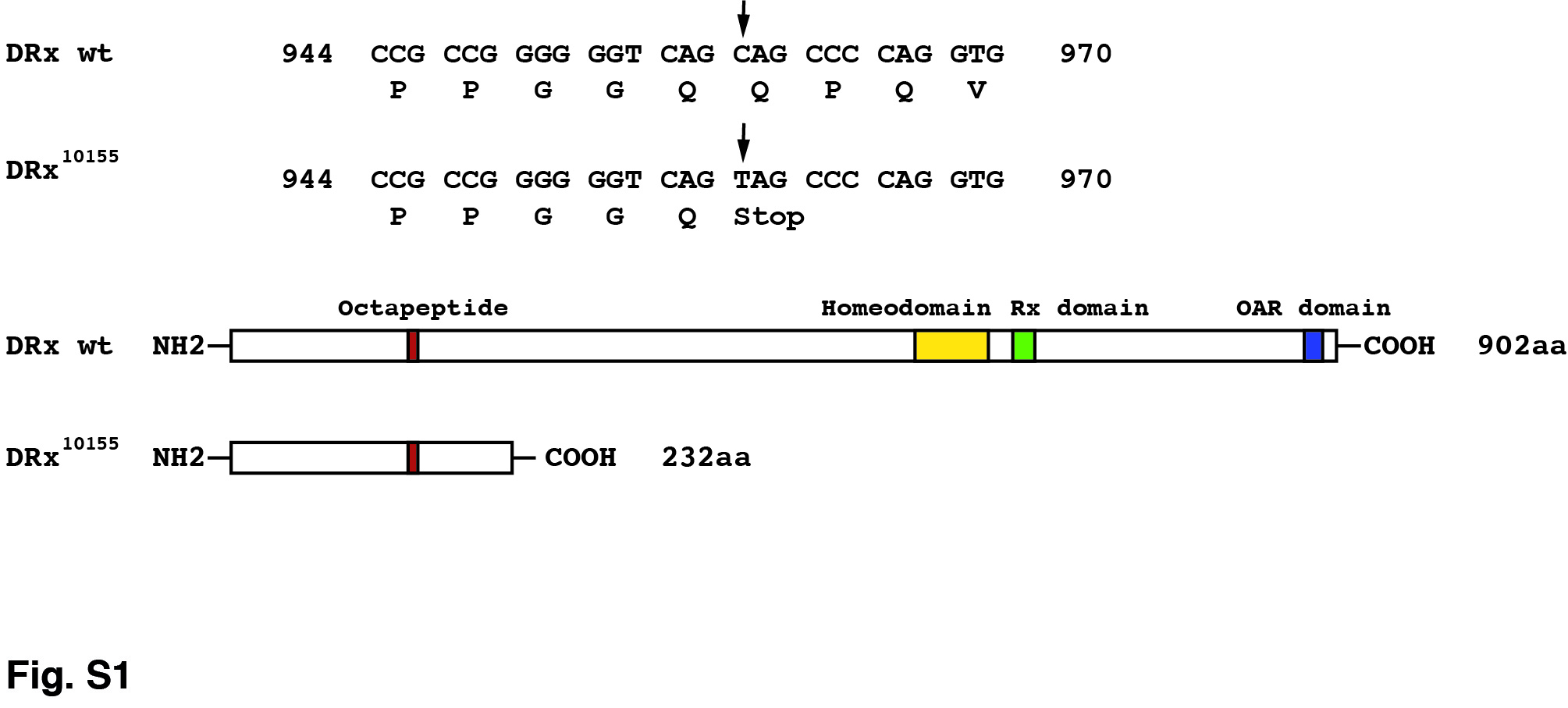

Supplement: Supplementary file 1 — Additional file 1: Figure S1. Molecular analysis of the DRx10155 allele. Nucleotide and amino acid sequences in wild-type (here DRx1 cDNA as an example) and mutant DNA. EMS induced a C to T transition in the coding region of DRx10155 leading to the formation of a stop codon. Schematic overview of the wild-type DRx protein with the localisation of the octapeptide (red), the homeodomain (yellow), the Rx domain (green) and the OAR domain (blue) in comparison to the truncated mutant protein of the DRx10155 allele. [file 41065_2021_210_MOESM1_ESM.jpg]

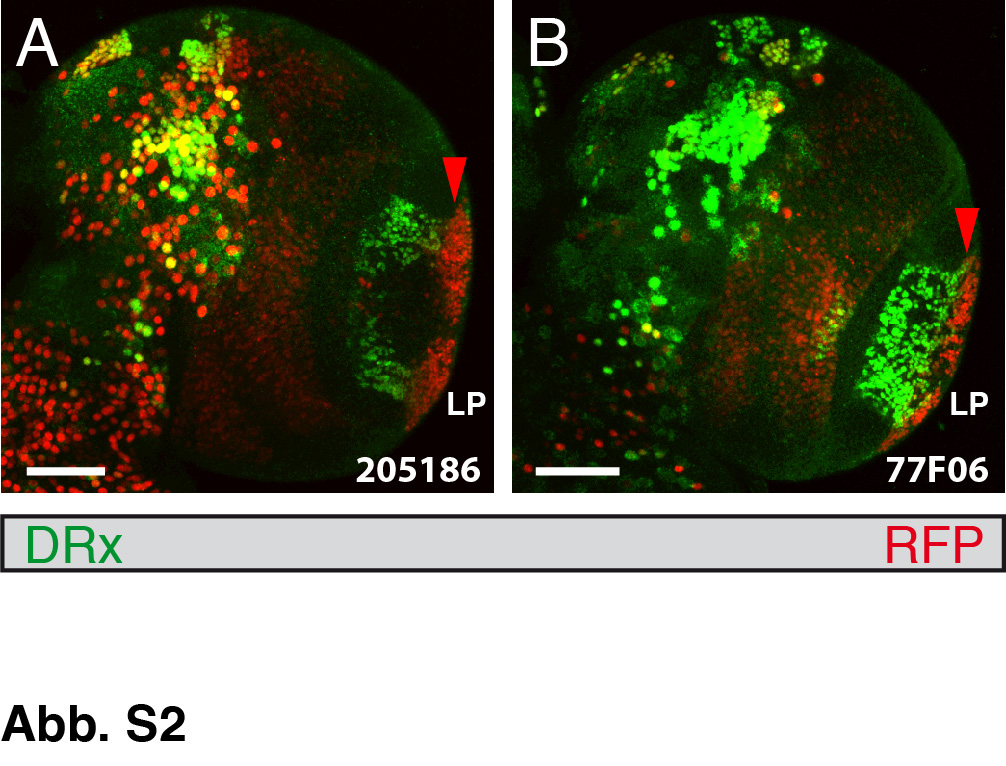

Supplement: Supplementary file 2 — Additional file 2: Figure S2. Expression of enhancer-Gal4 strains in the lobula. (A, B) Views of right hemispheres of Drosophila L3 larval brains. An anti-DRx antibody was used to visualize the nuclear DRx expression pattern in green, and enhancer-Gal4 driven UAS-H2B-mRFP1 expression showed the patterns generated by the various enhancers in red. The Gal4 strain numbers are indicated, and the red arrowheads indicate the regions where the enhancer expression is visible. LP, lobula plate. (Scale bar: 50 μm). [file 41065_2021_210_MOESM2_ESM.jpg]
